# The GABA_A_-Benzodiazepine Receptor Antagonist Flumazenil Abolishes the Anxiolytic Effects of the Active Constituents of *Crocus sativus* L. Crocins in Rats

**DOI:** 10.3390/molecules25235647

**Published:** 2020-11-30

**Authors:** Nikolaos Pitsikas, Petros A. Tarantilis

**Affiliations:** 1 Department of Pharmacology, Faculty of Medicine, School of Health Sciences, University of Thessaly, Biopolis, Panepistimiou 3, 415-00 Larissa, Greece; 2 Laboratory of Chemistry, Department of Food Science and Human Nutrition, School of Food and Nutritional Sciences, Agricultural University of Athens, 118-55 Athens, Greece; ptara@aua.gr

**Keywords:** crocins, flumazenil, anxiety, rat

## Abstract

Anxiety is a chronic severe psychiatric disorder. Crocins are among the various bioactive components of the plant *Crocus sativus* L. (Iridaceae) and their implication in anxiety is well-documented. However, which is the mechanism of action underlying the anti-anxiety effects of crocins remains unknown. In this context, it has been suggested that these beneficial effects might be ascribed to the agonistic properties of these bioactive ingredients of saffron on the GABA type A receptor. The current experimentation was undertaken to clarify this issue in the rat. For this research project, the light/dark and the open field tests were used. A single injection of crocins (50 mg/kg, i.p., 60 min before testing) induces an anti-anxiety-like effect revealed either in the light-dark or open field tests. Acute administration of the GABA_A_-benzodiazepine receptor antagonist flumazenil (10 mg/kg, i.p., 30 min before testing) abolished the above mentioned anxiolytic effects of crocins. The current findings suggest a functional interaction between crocins and the GABA_A_ receptor allosteric modulator flumazenil on anxiety.

## 1. Introduction

Anxiety is a serious psychiatric disease. Various forms of this psychiatric disorder, such as generalized anxiety disorder (GAD), specific phobias (agoraphobia, social phobia, etc.), post-traumatic stress disorder (PTSD), obsessive-compulsive disorder (OCD) and panic disorder have been described. Common features of all these disorders are temporary worry and exaggerated fear [1].

Although a conspicuous number of pharmacological approaches are actually used aiming to alleviate the symptoms of this psychiatric pathology [f.i., benzodiazepines, partial agonists of the serotonergic 5-HT_1A_ receptor, selective serotonin reuptake inhibitors (SSRIs)] different types of anxiety do not respond satisfactorily to these medications [2]. Additionally, these medications are often associated to severe side effects [3].

Based on the above, there is a mandatory necessity to unfold novel molecules for the therapy of this severe psychiatric disease [4]. Among the various alternative approaches for the treatment of anxiety symptoms, the involvement of the extracts of the stigmas of *Crocus sativus* L. (saffron) and its bioactive constituents as potential anti-anxiety agents has recently been proposed [5].

*Crocus sativus* L. (Iridaceae) is a plant cultivated in many countries all around the world including Iran, India, Italy, Spain and Greece. Its product is the well-known spice saffron. Saffron is the dried red stigmas of the flower. The main substances of saffron are crocins, picrocrocin and safranal. Crocins, glucosyl esters of crocetin, are water-soluble carotenoids and are responsible for its characteristic color. Picrocrocin, glycoside of safranal, is responsible for the bitter taste of the spice and is precursor of safranal. Safranal, the main component of the distilled essential oil, is a monoterpene aldehyde, responsible for its characteristic aroma [6,7].

The stigmas of *C. sativus* L. (saffron) are used in folk medicine as an anticatarrhal, eupeptic, expectorant and emmenagogue [8]. Contemporary preclinical pharmacological studies have demonstrated that saffron’s crude extracts and purified chemicals possess anti-tumor effects, display anti-inflammatory properties and counteract atherosclerosis and hepatic damage [8]. Additionally, the outcome of various preclinical and clinical studies suggest a promising effect of saffron and its bioactive constituents in different pathologies of the central nervous system including depression, schizophrenia, memory disorders and anxiety [5].

Specifically, crocins were found to display anxiolytic effects in different behavioral procedures assessing anxiety either in rats [9,10] or mice [11]. However, the mechanism of action underlying the anti-anxiety effects of crocins is not yet elucidated.

Consistent experimental evidence proposes that the anxiolytic effects of benzodiazepines are mediated by their agonistic action on the GABA_A_ receptor [12]. In this context, it has been observed that some other flavonoids isolated from plants express an affinity for the benzodiazepine binding site at the GABA_A_ receptor [13,14]. It has been reported that crocins enhance the anti-epileptic properties of diazepam [15] while the anti-convulsant action of safranal seems to be mediated by its agonistic action on the GABA_A_ receptor [16].

Based on the above, it can be hypothesized that the GABA_A_ receptor might be a potential target of anxiolytic effects of crocins. The current study was designed to examine this issue. Consequently, the anxiolytic-like effects of crocins in the rat were challenged with the benzodiazepine receptor antagonist, flumazenil. The light/dark box and open field tests were the behavioral paradigms used for this evaluation. The light/dark test is a behavioral procedure that is based on the innate aversion of rodents to strongly illuminated zones and the conflicting tendency of rodents to explore new spaces [17]. The open field test implies an encounter of the rodent with new open spaces and trigger behavioral and physiological reactions associated to anxiety [18].

## 2. Results

### 2.1. Experiment 1: Effects of Acute Administration of Crocins and Flumazenil on Rats’ Performance in the Light/Dark Test

**Figure 1:**
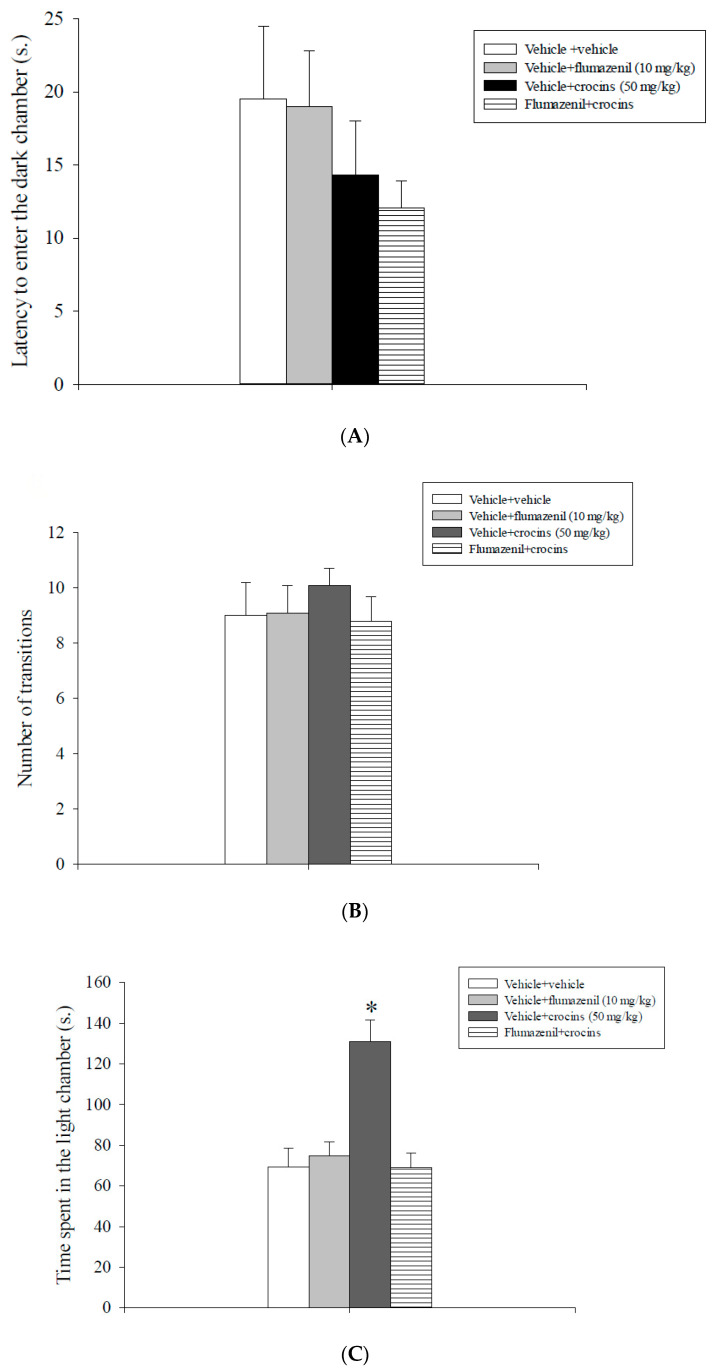
Light/dark box test. Crocins and flumazenil were injected intraperitoneally 60 and 30 min respectively before testing. The graphic illustrates the means ± S.E.M. of 8 rats per experimental group. (**A**) Latency to enter the dark chamber. (**B**) Number of transitions. (**C**) Time spent in the light chamber. * *p* < 0.05 vs. all the other groups.

Data are illustrated in Figure 1. Analysis of the first entry into to the dark chamber (Figure 1A) and the number of transitions between the two compartments data (Figure 1B) did not evidence a statistically significant main effect either of flumazenil or of crocins or a statistically significant interaction between crocins and flumazenil. Analysis of the total time spent in the light chamber data revealed a statistically significant main effect of flumazenil [F(1,31) = 10.24, *p* = 0.003], of crocins [F(1,31) = 10.52, *p* = 0.003] and a significant flumazenil x crocins interaction [F(1,31) = 15.02, *p* < 0.001]. The post-hoc analysis conducted on these data showed that rats treated with vehicle + crocins (50 mg/kg) spent more time in the lit chamber of the apparatus with respect to the vehicle + vehicle, flumazenil (10 mg/kg) + vehicle and flumazenil (10 mg/kg) + crocins (50 mg/kg) groups (*p* < 0.05, Figure 1C).

### 2.2. Experiment 2: Effects of Acute Administration of Crocins and Flumazenil on Rats’ Performance in the Open Field Test

Data are illustrated in Table 1. The effects of acute treatment either with flumazenil or crocins did not affect the number of squares crossed, the rearing and grooming episodes. Interestingly, a main effect of flumazenil [F(1,31) = 12.5, *p* = 0.001], of crocins [F(1,31) = 9.7, *p* = 0.004] and a statistically significant interaction between flumazenil and crocins [F(1,31) = 15.65, *p* < 0.001] was evidenced with regard to the time consumed in the central zone of the arena. The post-hoc comparisons showed that rats that received crocins (50 mg/kg) + vehicle spent more time in the central area of the open field apparatus compared to all the other treatment groups (*p* < 0.05).

## 3. Discussions

The light/dark test has been shown to reliably predict the anxiolytic- and anxiogenic-like effects of drugs in rodents [17]. This test has the advantages of being quick and easy to use without prior training of the animals and neither food nor water deprivation is required [19]. Transitions in this test are considered an index of activity/exploration because habituation over time is seen with this measure, whereas the time spent in each compartment reflects aversion/attraction [20].

The open field test is a typical behavioral paradigm of anxiety evaluating neophobia. In this behavioral procedure either mice or rats usually manifest fear for open spaces. Therefore, the amount of time consumed in the central zone of the apparatus reflects an anxiety state [18].

The objective of the current study was to examine whether or not the GABA_A_-benzodiazepine receptor is involved in the anti-anxiety properties of crocins.

In agreement with previous results of ours, crocins induced anxiolytic-like effects in rats [9,10]. In particular, crocins (50 mg/kg) administered 60 min before testing, in rats augmented the time consumed in the aversive (lit) compartment of the apparatus (light/dark box) and the time consumed in the central zone of the open field arena as compared with all the other experimental groups. It is the first time, to our knowledge, that the anti-anxiety effects of crocins were observed in the open field test. Further, the current findings are in line with prior reports in which the aqueous extracts of saffron and crocin were found to reduce stress-induced anorexia [11] in mice and diminish anxiety symptoms assessed in the elevated plus maze test and freezing behavior in rats [21].

The GABA_A_-benzodiazepine receptor antagonist flumazenil blocked the anxiolytic effects of crocins in both anxiety procedures tested. Flumazenil on its own was inactive either in the light/dark or in the open field test. The results here exposed propose that the anxiolytic effects of crocins may be mediated by their interaction with the benzodiazepine binding site on the GABA_A_ receptor.

Chemicals that influence general activity may alter rodents’ performance in anxiety tests because of changes in motoric activity that are unrelated to any anxiogenic- or anxiolytic effects of the compound. The results here presented cannot be ascribed to a potential action exerted by both crocins and flumazenil on general activity because the number of transitions between the two different compartments in the light/dark box test and the number of squares crossed and number of rearing events registered in the open field test did not vary among the different treatment groups.

Our results are in contrast with a recent study in which flumazenil was found unable to reverse the effects of crocins on anxiety [22]. Important differences however, underlie this discrepancy. In the present study, crocins (50 mg/kg) were injected 60 min before testing and displayed their anxiolytic profile in agreement with previous studies [9,10]. Further, flumazenil was injected at the dose of 10 mg/kg, 30 min before testing. On the contrary, in the study by Ceremuga and colleagues [22] the treatment schedule of compounds was different to that used in our study. Specifically, crocins (50 mg/kg, 30 min before testing) failed to induce an anti-anxiety effect while flumazenil was administered at a lower dose and at different time point (3 mg/kg, 10 min before testing) and did not affect crocins performance.

It is well-documented that flumazenil counteracted the anxiolytic effects also of non-benzodiazepine compounds (f.i., 5-HT1_A_ receptor agonists and 5-HT_3_ receptor antagonists). This suggests that the GABA_A_-benzodiazepine receptor may be a common downstream component of different neurochemical systems modulating anxiety states [23].

The observed reversal of the effects of flumazenil on crocins anxiolytic effects corroborates the hypothesis of crocins acting directly or indirectly on the GABA_A_-benzodiazepine receptor. A direct interaction of crocins with the GABA_A_ receptor complex might involve their action on Cl^-^ conductance. An indirect action of crocins might reside on the modulation of metabolism or regulation of GABA release. Research is mandatory in order to evaluate this hypothesis.

The GABA type A receptor is a pentameric ligand-gated chloride channel constituted of distinct classes of subunits (α1–α6, β1–β3, γ1–γ3, δ, ε, θ, π and ρ1–ρ3). This receptor is a molecular target for various classes of benzodiazepine-site ligands. It has been suggested that binding to the α2 subunit is crucial for the anxiolytic effects of benzodiazepines [24]. Thus, by utilizing appropriate ligands it will be of interest to investigate whether or not the anxiolytic effects of crocins might be mediated by their interaction with the α2 subunit of the GABA_A_ receptor. In line with the above, additional studies should be performed aiming to elucidate if the anti-anxiety effects displayed by crocins can be mediated also by other types of receptors related to GABAergic, serotonergic or to glutamatergic system.

In summary, the present results suggest that the GABA_A_-benzodiazepine receptor complex might modulate crocins’ beneficial effects on anxiety. The current findings also indicate a functional interaction between crocins and the benzodiazepine ligand site.

## 4. Materials and Methods

### 4.1. Drugs

Crocins used in the current experimentation were derived from the same batch of plant material (saffron) and the same purification procedure, extraction and separation. Our plant material was kindly offered by the Cooperative of Saffron, Krokos, Kozani, Greece.

Crocins were isolated from the red dried stigmas of *C. sativus* using a slightly modified method described previously [25]. They were purified from stigmas after successive and exhaustive extraction by: (a) petroleum ether 40–60 °C; (b) diethyl ether (Et_2_O) and (c) methanol (MeOH) 80% using ultrasound assisted extraction. The ultrasound extraction was performed in a Sonorex, Super RK 255H type (300 mm × 150 mm × 150 mm internal dimensions) ultrasound water bath (indirect sonication), at the fixed frequency of 35 kHz. The temperature of the sonicated water was 25 °C. Procedures (a) and (b) took place in order for the stigmas to be free the final extract from the presence of unwanted compounds such as lipids, safranal and picrocrocin. The methanol extract after evaporation (condensed to dryness) under vacuum at room temperature, provided crocins, which are dark red powder residue. Crocins are unusual water-soluble carotenoids (glucosyl esters of crocetin). Τhe chemical profile of crocins has been well documented in previous studies [7,26,27,28] and we evaluated the quality of the fresh prepared extract used in this study. The major component is a digentiobiosyl ester of crocetin [29]. The purity of crocins was 85% (by HPLC). Crocins were dissolved in saline (NaCl 0.9%). The dose of crocins (50 mg/kg) which induced the highest anti-anxiety properties under to our experimental conditions was selected based on previous findings [9,10]. Specifically, in a previous study designed to test at which concentration crocins might display an anti-anxiety it has been demonstrated that crocins were effective as anxiolytic agents at 50 but not at 15 or 30 mg/kg [9].

Flumazenil (Sigma, St. Louis, MO, USA) was dissolved in saline containing 0.1% Tween 80. The dose of flumazenil (10 mg/kg) was selected based on previous studies [30,31] and our unpublished observations. In those studies [30,31] flumazenil reversed the anxiolytic effects of different potential anti-anxiety agents as are the g-hydroxybutyrate, a product of GABA metabolism and MGS0039, a selective group II metabotropic glutamate receptor antagonist.

All drug solutions were freshly prepared on the day of testing and were administered intraperitoneally (i.p.) in a volume of 1 mL/kg. For all studies, control animals received isovolumetric amounts of the specific vehicle solutions.

### 4.2. Animals

Independent groups of naive male (3-month-old) Wistar rats (Hellenic Pasteur Institute, Athens, Greece) weighing 250–300 g were used. The animals were housed in Makrolon cages (47.5 cm length × 20.5 cm height × 27 cm width), three per cage in a regulated environment (21 ± 1 °C; 50–55% relative humidity; 12-h/12-h light/dark cycle, lights on at 07.00 h) with free access to food and water.

The procedures that involved animals and their care were in accordance with international guidelines and national (Animal Act, P.D. 160/91) and international laws and policies (EEC Council Directive 86/609, JL 358, 1, 12 December 1987). Experiments were approved by the local committee (Prefecture of Larissa, Greece, protocol number 255200/1 October 2020).

### 4.3. Behavior

#### 4.3.1. Light/Dark Test

The light/dark box apparatus consisted of a wooden box (48 cm length × 24 cm height × 27 cm width) divided into two equal-size chambers by a barrier that contained a doorway (10 cm height × 10 cm width). One of the chambers was painted black and was covered with a lid and the other chamber was painted white and illuminated with a 60-W light bulb placed 40 cm above the upper edge of the apparatus. Testing was conducted in agreement to a prior report [32]. In brief, on the test day, animals were moved to the obscured test room and remained in their home cages for 2 h. Subsequently, the rats were positioned in the middle of the lit chamber, facing away from the dark chamber. Animals were allowed to freely move the test apparatus for 5 min. The latency to enter (with all four paws) the dark chamber, number of transitions and time spent in the light and dark compartments were registered.

#### 4.3.2. Open Field Test

The test apparatus consisted of a dark arena made of Plexiglas (70 cm length × 50 cm height × 70 cm width). The open field was divided-by black lines-into 16 squares of 17.5 × 17.5 cm^2^. The central four squares were defined as the central area, in which rats’ behavior was considered as a measure of anxiety [18]. The test was conducted in agreement to previous reports [32,33]. On the test day, the rats were moved to the dimly illuminated (20 lux) test room and remained their home cages for 2 h. Subsequently, each rat was positioned in the same corner of the apparatus and its behavior was registered for 5 min. The parameters recorded were: (a) the total time spent in the central area of the open field arena as defined by all forepaws being in the central four squares of the apparatus, (b) the number of squares crossed (which reflects horizontal activity), (c) the number of rearing episodes (which reflects vertical activity, defined as raising both forepaws above the floor while balancing on hind limbs) and (d) the duration of self-grooming episodes.

### 4.4. Experimental Protocol

Daily testing was carried out between 9:00 AM and 3:00 PM during the light phase of the light/dark cycle. To avoid the presence of olfactory traces, all the apparatuses (light/dark box and open field apparatus) were intensively cleaned with 20% ethanol and then wiped with dry paper after each animals’ performance.

Animals’ behavior in the light/dark and open field tests was video-recorded. Data evaluation was performed by an experimenter who was not involved in the experimental protocol.

#### 4.4.1. Experiment 1: Effects of Acute Challenge with Crocins and Flumazenil on Animals’ Performance in the Light/Dark Test

Animals were randomly divided into four experimental groups with 8 rats per group as follows: vehicle (NaCl 0.9%) + vehicle (NaCl 0.9% containing 0.1% Tween 80); vehicle (NaCl 0.9% containing 0.1% Tween 80) + flumazenil (10 mg/kg); vehicle (NaCl 0.9%) + crocins (50 mg/kg) and flumazenil (10 mg/kg) + crocins (50 mg/kg). Control rats were treated with the vehicle 60 and 30 min i.p. respectively before testing. Crocins and flumazenil were injected 60 and 30 min respectively before testing.

#### 4.4.2. Experiment 2: Effects of Acute Challenge with Crocins and Flumazenil on Animals’ Performance in the Open Field Test

The same experimental design used in experiment 1 was applied for experiment 2. 

### 4.5. Statistical Analysis

Data are expressed as mean ± S.E.M. Data were analyzed using the two-way analysis of variance (ANOVA). The factors were flumazenil and crocins. Post-hoc comparisons between treatment means were made with the Tukey’s post-hoc test. Values of *p* < 0.05 were considered statistically significant.

## Figures and Tables

**Table 1:** Effects of acute treatment with crocins and flumazenil on rats’ performance in the open field test (n = 8 rats per group).

Treatment	Number of Squares Crossed	Number of Rearings	Time Spent in the Central Zone (s.)	Grooming Duration (s.)
Vehicle + vehicle	96 ± 5.5	29.4 ± 4	7.8 ± 2.3	10.8 ± 2.1
Flumazenil (10 mg/kg) + vehicle	107.3 ± 6.3	32 ± 1.4	9.3 ± 2.1	9.4 ± 1.7
Crocins (50 mg/kg) + vehicle	94.4 ± 4	29 ± 1.5	21.1 ± 1.7 *	7.8 ± 0.8
Flumazenzil + crocins	99.6 ± 4.9	32.8 ± 2.5	8.5 ± 0.7	9.9 ± 0.5

Crocins and flumazenil were injected intraperitoneally 60 or 30 min before testing respectively. The values are mean ± SEM. * *p* < 0.05 vs. all the other groups.
